# A hemorheological perspective on coronary microvascular dysfunction: Association of erythrocyte parameters with angiography-derived coronary microcirculatory resistance

**DOI:** 10.1371/journal.pone.0345562

**Published:** 2026-03-25

**Authors:** Abdulrahman AlQazzaz, Yanfeng Lu, Jasmine Yimeng Bao, Gary S. Mintz, Jiahao Feng, Yong Zhang, Shanshan Gao, Qiang Song, Feifei Ning, Hytham H. Al-Samawi, Mohsen Al-Manj, Mohammed Al-Asadi, Xin Huang, Ning Guo

**Affiliations:** 1 Department of Cardiology, The First Affiliated Hospital of Xi’an Jiaotong University, Xi’an, Shaanxi, China; 2 Sidney Kimmel Medical College, Thomas Jefferson University, Philadelphia, Pennsylvania, United States of America; 3 Cardiovascular Research Foundation, New York, New York, United States of America; 4 Department of Cardiology, The Second Affiliated Hospital of Xi’an Jiaotong University, Xi’an, Shaanxi, China; Pescara General Hospital, ITALY

## Abstract

**Background:**

Coronary microvascular dysfunction (CMD) contributes to myocardial ischemia in patients both without obstructive coronary artery disease (e.g., MINOCA/INOCA) and in those with co-existing epicardial stenosis. While its etiology includes structural and functional causes. hematologic parameters have been linked to cardiovascular outcomes. However, the relationship between red blood cell (RBC) markers and microvascular resistance remains poorly characterized. We aim to evaluate whether RBC parameters are correlated with the angiography-derived index of microcirculatory resistance (angio-IMR).

**Methods:**

This retrospective study evaluated the association between red blood cell (RBC) parameters and angio-IMR in patients with intermediate coronary artery disease (30%−70% stenosis). Data were analyzed from 604 patients, comprising 733 lesions; red blood cell parameters were obtained during hospitalization prior to angiography. Coronary microcirculatory resistance was derived using the AngioPlus mQFR system. Multivariable linear regression models adjusted for confounders. Subgroup analyses assessed effect modification by diabetes status, and sensitivity analyses excluded hematocrit outliers, and analyses stratified by vessel (left anterior descending (LAD), left circumflex (LCx), right coronary artery (RCA)).

**Results:**

Higher RBC, Hct and Hgb were independently associated with elevated angio-IMR after full adjustment (RBC: β = 0.182, *P* < 0.001; Hct: β = 0.019, *P* < 0.001; Hgb: β = 0.006, *P* < 0.001). Results remained robust after excluding Hct outliers (β = 0.020, *P* < 0.001) and consistent across diabetic (*P* = 0.007) and non-diabetic subgroups (*P* = 0.012). The association was significant in non-LAD vessels (Hct: β = 0.022, P < 0.001) but not in LAD lesions (Hct: β = 0.008, P = 0.357).

**Conclusion:**

Elevated RBC parameters are independently associated with increased microcirculatory resistance, particularly in non-LAD vessels. These findings suggest that RBC parameters may serve as clinically relevant markers of microvascular dysfunction, warranting further investigation into their prognostic and therapeutic implications.

## Introduction

Coronary microvascular dysfunction (CMD) is a complex pathophysiological condition that can lead to significant clinical consequences including myocardial infarction with non-obstructive coronary arteries (MINOCA) [1] and ischemia with non-obstructive coronary arteries (INOCA) [2] as well as in patients with obstructed coronary arteries. The underlying etiology is multifactorial and can be broadly categorized into structural and functional abnormalities [3,4].

Structural causes include lumen obstruction, perivascular fibrosis, microvascular invasion and remodeling [5], platelet activation [6], or microembolization of thrombotic material from the proximal coronary artery [7], as well as some conditions such as left ventricular hypertrophy which may elevate intramyocardial pressure leading to increased resistance to blood flow [8]. Functional contributors to CMD include impaired vasomotor responses, principally endothelial dysfunction in the coronary microcirculation leading to impaired flow-mediated dilation [9,10], as well as pathological vasoconstriction manifesting as microvascular spasm [11].

CMD has been associated with multiple risk factors, including chronic inflammatory diseases [12] such as systemic lupus erythematosus (SLE) and rheumatoid arthritis [13], as well as traditional cardiovascular risk factors (advanced age, hypertension, dyslipidemia, diabetes mellitus, and smoking) [14–17]. However, as demonstrated by the WISE study, these established factors account for less than 20% of the observed variability in coronary microvascular reactivity, as measured by coronary flow velocity reserve response to intracoronary adenosine (CFVR Ado) among women with suspected ischemia [18]. Given these limitations we investigated novel hematologic factors that may contribute to microvascular impairment.

Previous investigations have established associations between erythrocyte parameters and cardiovascular outcomes. The landmark Finnish cohort study by Kunnas et al. (2009) first demonstrated this relationship [19], with subsequent studies confirming significant correlations between hematocrit levels and cardiac morbidity [20–22] as well as hemoglobin concentrations and cardiovascular health [23–25], These findings highlight the critical need to explore the association between blood rheology markers and microvascular dysfunction—an area that remains understudied.

To investigate this relationship, angiography-derived microcirculatory resistance (angio-IMR) was selected for this study based on its validated correlation with invasive wire-based index of microcirculatory resistance (IMR) measurements [26–28]. This approach offers significant clinical advantages, including being less invasive, more cost-effective, and technically simpler to perform while maintaining comparable diagnostic accuracy [29–31] even when based on a single angiographic view [32]. Furthermore, angio-IMR provides prognostic value for long-term outcomes and microvascular obstruction, as demonstrated in previous studies [33–36].

Therefore, we sought to determine the association between erythrocyte parameters and angiography-derived microcirculatory resistance in a cohort of patients with intermediate coronary stenosis.

## Methods

### Study design and population

This retrospective cohort study evaluated the association between red blood cell (RBC) parameters and Angio-IMR in patients with coronary artery disease (CAD). Data were obtained during (5 June 2024–10 December 2024) from the First Affiliated Hospital of Xi’an Jiaotong University (Xi’an, China) database, comprising 733 lesions (10 May 2023–30 June 2023) initially collected for a quantitative flow ratio (QFR) grey-zone study. All coronary angiograms were performed in an elective setting for the diagnostic evaluation of stable ischemic symptoms or suspected coronary artery disease.

Inclusion criteria comprised: (1) patients meeting AngioPlus standard criteria for adequate monoplane QFR (mQFR) analysis (optimal contrast filling and minimal vessel overlap in a single angiographic projection) and (2) intermediate stenosis (30–70% diameter stenosis) in at least one major epicardial vessel. Exclusion criteria were: (1) insufficient angiographic quality for mQFR/IMR analysis (e.g., poor contrast opacification, foreshortening), (2) stented target vessels (to avoid flow artifacts), (3) chronic total occlusion (CTO) or retrograde collateral filling, (4) ostial left main or right coronary artery lesions (due to mQFR technical limitations), and (5) prior coronary artery bypass grafting (CABG) involving the target vessel.

Hematological variables included Red Blood Cells count (RBC), Hematocrit (Hct) and Hemoglobin (Hgb). extracted from complete blood count (CBC) results obtained after admission and before angiography. Other blood parameters included Mean Corpuscular Volume (MCV), Mean Corpuscular Hemoglobin (MCH), Mean Corpuscular Hemoglobin Concentration (MCHC), Red Cell Distribution Width – Coefficient of Variation (RDW CV), Red Cell Distribution Width – Standard Deviation (RDW SD), Platelets (PLT), Platelet Distribution Width (PDW), White Blood Cells (WBC). Clinical demographics (included age, sex, BMI), comorbidities (hypertension, diabetes mellitus, dyslipidemia), medications (prior statins), and angiographic characteristics (stenosis severity, lesion length, vessel location). To ensure the validity of both the measured hematologic parameters and the coronary microcirculatory assessment, no patient in the cohort received a packed red blood cell transfusion in the 48 hours preceding either the blood sample collection for complete blood count analysis or the index coronary angiography.

Complete blood count analysis was performed using a Sysmex XN-9000 automated hematology analyzer (Sysmex Corporation, Kobe, Japan), and Angio-IMR was calculated with the AngioPlus software (AngioPlus Galley 2.1, Pulse Medical, Shanghai, China). A single angiographic projection with minimal foreshortening and optimal contrast filling was selected for computational fluid dynamics (CFD)-based simulation of hyperemic flow. IMR was calculated as:


$$IMR=P_{d}\times T_{mn}$$

where *P*_*d*_ = distal pressure and *T*_*mn*_ = mean transit time under simulated hyperemia. mQFR was co-registered as a secondary measure of functional stenosis severity.

### Statistical analysis

The **primary analysis** employed multiple linear regression models with sequential adjustment for potential confounders. Model 1 assessed the association between RBC parameters and angiography-derived IMR, adjusted for other blood parameters. Model 2 further adjusted for demographic factors (age, sex, BMI), while Model 3 incorporated comorbidities (diabetes mellitus, hypertension, hypercholesterolemia and prior statins). The final fully adjusted model (Model 4) included angiographic severity (diameter stenosis (DS), lesion length) in addition to all previous covariates.

**Secondary analyses** were conducted to explore potential effect modification and robustness of the findings. Given the known impact of diabetes mellitus on RBC deformability, subgroup analysis stratified by diabetes status was performed. Sensitivity analysis excluded extreme Hct outliers (<30% or >50%) to mitigate potential hemodilution or polycythemia-related bias. To account for hemodynamic and anatomical differences between coronary vessels, sensitivity analyses were conducted by stratifying lesions into LAD versus non-LAD (LCx + RCA) subsets, with Model 4 replicated in each group. Additionally, vessel-specific analyses were performed by applying Model 4 separately to LAD, LCx, and RCA lesions to evaluate potential vessel-dependent associations.

Statistical significance was defined as a two-tailed P < 0.05. The normality of continuous variables was assessed using the Shapiro-Wilk test. For multivariate models, multicollinearity was assessed using variance inflation factors (VIF < 5 considered acceptable). Leukocyte subtypes were examined in exploratory analyses but were not included in the final models due to non-significant associations with the outcome and high multicollinearity with total white blood cell count (VIF > 10) (S2 Table). Model assumptions were verified through residual diagnostics. Sensitivity analyses excluding outliers were performed to ensure the stability of the results. For the IMR assessment, mQFR was selected due to its validated accuracy in deriving IMR from a single angiographic projection, reducing reliance on multi-angle acquisitions while maintaining diagnostic precision [32]. Lesions with suboptimal contrast timing or vessel overlap were excluded to ensure analytical reliability.

Analyses were performed using SPSS v27 (IBM, Armonk, NY). Continuous variables were reported as mean ± SD (normally distributed); categorical variables as frequencies (%).

This study was conducted in accordance with the Declaration of Helsinki and was approved by the Ethics Committee of The First Affiliated Hospital of Xi’an Jiaotong University. The requirement for informed consent was waived by the ethics committee due to the retrospective nature of the study.

## Results

### Population characteristics

The study included 733 lesions from 604 patients with CAD (age 64.5 ± 10.4 years; 70.9% male) (Table 1). Comorbidities were prevalent, with hypertension (63.1%), hypercholesterolemia (38.9%), and diabetes mellitus (32.9%) frequently observed. Hematologic parameters were within normal ranges (Hct: 41.60 ± 4.97%; Hgb: 135.88 ± 17.21 g/L; RBC: 4.39 ± 0.56 × 10¹²/L). Angiographic data revealed moderate stenosis severity (DS%: 45.36 ± 7.63) and lesion length of 29.2 ± 15.9 mm.

**Table 1:** Population characteristics.

*Patient characteristics (n = 604)*	
Age (years)	64.42 ± 10.48
Male sex, n (%)	428 (70.9%)
BMI (kg/m²)	24.62 ± 3.24
Diabetes Mellitus, n (%)	199 (32.9%)
Hypertension, n (%)	381 (63.1%)
Hypercholesterolemia, n (%)	235 (38.9%)
Prior statins, n (%)	333 (55.1%)
Blood Parameters	
RBC (×10¹²/L)	4.39 ± 0.56
Hct (%)	41.59 ± 4.97
Hgb (g/L)	135.88 ± 17.21
MCV (fL)	94.79 ± 5.02
MCH (pg)	30.95 ± 1.96
MCHC (g/L)	326.45 ± 8.35
RDW CV (%)	13.33 ± 4.48
RDW SD (fL)	44.30 ± 3.38
PLT (×10⁹/L)	205.81 ± 58.43
PDW (fL)	16.12 ± 1.28
WBC (×10⁹/L)	6.51 ± 2.22
Number of Lesions per patient	
1 Lesion	483 (80%)
2 Lesions	113 (18.7%)
≥ 3 Lesions	8 (1.3%)
***Lesion characteristics (n = 733)***	
Simulated hyperemic MR	2.38 ± 0.58
DS%	45.36 ± 7.63
Lesion length (mm)	29.19 ± 15.85
Lesion location, n (%)	
LAD	228 (39.3%)
LCx	250 (34.1%)
RCA	195 (26.6%)

BMI: body mass index; DS%: diameter stenosis percentage; Hct: hematocrit; Hgb: hemoglobin; LAD: left anterior descending artery; LCx: left circumflex artery; MCH: mean corpuscular hemoglobin; MCHC: mean corpuscular hemoglobin concentration; MCV: mean corpuscular volume; MR: microcirculatory resistance; PDW: platelet distribution width; PLT: platelets; RBC: red blood cell count; RCA: right coronary artery; RDW CV: red cell distribution width – coefficient of variation; RDW SD: red cell distribution width – standard deviation; WBC: white blood cells.

### Stratification by IMR tertiles

Patients were stratified into low (<2.07), medium (2.07–2.6), and high (>2.6) IMR tertiles (Table 2). The high IMR group exhibited significantly higher RBC parameters (RBC: 4.48 ± 0.53 vs. 4.30 ± 0.58, *P* = 0.002; Hct: 42.64 ± 4.51 vs. 40.55 ± 5.25, *P* < 0.001; Hgb: 139.7 ± 15.7 vs. 131.8 ± 18.1 g/L, *P* < 0.001) compared to the low IMR group. Male sex was more prevalent in the high IMR group (76.3% vs. 66%, *P* = 0.030), while lesion length was inversely associated with IMR (33.84 ± 16.59 mm in low IMR vs. 25.24 ± 14.50 mm in high IMR, *P* < 0.001).

**Table 2:** Comparison of Population Characteristics Across IMR Tertile Groups.

	Low IMR (<2.07) n = 244	Medium IMR (2.07–2.6) n = 244	High IMR (≥ 2.6) n = 245	Test Statistic*	*P value*
**Age (years)**	65 ± 10.4	64.7 ± 10.1	63.7 ± 10.8	2.080	0.353
**Male sex, n (%)**	161 (66%)	166 (68%)	187 (76.3%)	7.005	**0.030**
**BMI (kg/m²)**	24.7 ± 3	24.3 ± 3.2	24.8 ± 3.4	2.634	0.268
**Diabetes mellitus, n (%)**	81 (33.5%)	88 (36.4%)	73 (30.2%)	2.095	0.351
**Hypertension, n (%)**	153 (63.2%)	167 (69%)	151 (62.4%)	2.756	0.252
**Hypercholesterolemia, n (%)**	111 (45.9%)	86 (35.5%)	93 (38.4%)	5.730	0.057
**Prior statins, n (%)**	142 (58.7%)	135 (55.8%)	123 (50.8%)	3.084	0.214
**Blood Parameters**					
**RBC (×10¹²/L)**	4.30 ± 0.58	4.40 ± 0.56	4.48 ± 0.53	11.984	**0.002**
**Hct (%)**	40.55 ± 5.25	41.62 ± 4.88	42.64 ± 4.51	21.433	**<0.001**
**Hgb (g/L)**	131.8 ± 18.1	135.9 ± 16.6	139.7 ± 15.7	26.080	**<0.001**
**MCV (fL)**	94.3 ± 4.9	94.7 ± 5.0	95.3 ± 5.0	4.278	0.118
**MCH (pg)**	30.65 ± 1.99	30.94 ± 1.92	31.23 ± 1.93	13.258	**0.001**
**MCHC (g/L)**	324.8 ± 8.8	326.4 ± 7.9	327.5 ± 8.1	14.324	**<0.001**
**RDW CV (%)**	13.7 ± 6.9	13.1 ± 0.8	13.0 ± 0.9	5.729	0.057
**RDW SD (fL)**	44.42 ± 3.30	44.45 ± 3.53	44.20 ± 3.12	0.797	0.671
**PLT (×10⁹/L)**	209.2 ± 59.1	203.7 ± 56.3	206.0 ± 57.6	2.093	0.351
**PDW (fL)**	16.14 ± 1.17	16.15 ± 1.16	16.08 ± 1.33	4.124	0.127
**WBC (×10⁹/L)**	6.63 ± 2.46	6.47 ± 2.17	6.55 ± 2.15	0.586	0.746
**Angiographic data**					
**Simulated hyperemic MR**	1.77 ± 0.20	2.32 ± 0.15	3.04 ± 0.37	650.698	<0.001
**DS%**	45.58 ± 7.26	45.02 ± 7.71	45.47 ± 7.92	0.824	0.662
**Lesion length (mm)**	33.84 ± 16.59	28.51 ± 15.24	25.24 ± 14.50	38.519	**<0.001**

* Continuous variables were compared using Kruskal-Willis H while categorical variables were compared using Pearson Chi-Square.

BMI: body mass index; DS%: diameter stenosis percentage; Hct: hematocrit; Hgb: hemoglobin; MCH: mean corpuscular hemoglobin; MCHC: mean corpuscular hemoglobin concentration; MCV: mean corpuscular volume; MR: microcirculatory resistance; PDW: platelet distribution width; PLT: platelets; RBC: red blood cells count; RDW CV: red cell distribution width – coefficient of variation; RDW SD: red cell distribution width – standard deviation; WBC: white blood cells.

### Unadjusted analysis

**Fig 1:**
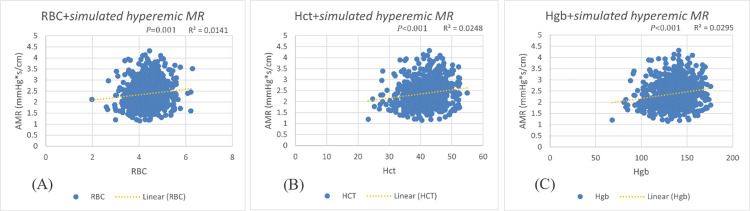
Unadjusted correlation between simulated hyperemic microcirculatory resistance and(A) RBC (B) Hct (C) Hgb.

Scatter plot analyses revealed significant positive correlations between simulated hyperemic MR and all measured RBC parameters. Hemoglobin demonstrated the strongest association (Hgb; r = 0.172, *P* < 0.001), followed by hematocrit (Hct; r = 0.157, *P* < 0.001) and red blood cell count (RBC; r = 0.119, *P* < 0.001) (Fig 1). The linear relationships were consistently maintained across the physiological ranges of each parameter, with no evidence of threshold effects. These unadjusted associations suggested that higher erythrocyte mass and hemoglobin content were proportionally associated with increased coronary microcirculatory resistance, independent of other clinical or angiographic factors.

### Primary regression analysis

Unadjusted analyses demonstrated significant associations between simulated hyperemic MR and RBC parameters (RBC: β = 0.123, *P* = 0.001; Hct: β = 0.018, *P* < 0.001; Hgb: β = 0.006, *P* < 0.001). These associations remained highly significant (*P* < 0.001 for all) through sequential multivariate adjustment: first for other blood parameters (Model 1), then additionally for demographics (Model 2), comorbidities (Model 3), and finally angiographic severity (Model 4). In the fully adjusted model, IMR maintained strong independent associations with RBC parameters (RBC: β = 0.182, 95% CI 0.090–0.274; Hct: β = 0.019, 0.009–0.028; Hgb: β = 0.006, 0.003–0.009; all *P* < 0.001) (Tables 3 and 4).The stability of effect sizes and persistent statistical significance across all models indicated these hematologic measures were robust independent predictors of coronary microcirculatory resistance, unaffected by potential clinical or angiographic confounders.

**Table 3:** Association of erythrocyte parameters with simulated hyperemic MR across sequential regression models.

Variable	Unadjusted β (95% CI)	Model 1 β (95% CI)	Model 2 β (95% CI)	Model 3 β (95% CI)	Model 4 β (95% CI)
***RBC***	.123 (0.048 to 0.198) ***P* = 0.001**	.187 (0.105 to 0.269) ***P* < 0.001**	.170 (0.075 to 0.265) ***P* < 0.001**	.168 (0.073 to 0.263) ***P* < 0.001**	.182 (0.090 to 0.274) ***P* < 0.001**
***Hct***	.018 (0.010 to 0.027) ***P* < 0.001**	.019 (0.010 to 0.027) ***P* < 0.001**	.017 (0.007 to 0.027) ***P* < 0.001**	.017 (0.007 to 0.027) ***P* < 0.001**	.019 (0.009 to 0.028) ***P* < 0.001**
***Hgb***	.006 (0.003 to 0.008) ***P* < 0.001**	.006 (0.004 to 0.008) ***P* < 0.001**	.006 (0.003 to 0.006) ***P* < 0.001**	.006 (0.003 to 0.009) ***P* < 0.001**	.006 (0.003 to 0.009) ***P* < 0.001**

*-Model 1: Adjusted for other blood parameters.*

*-Model 2: Model 1 + (age, sex and BMI).*

*-Model 3: Model 2 + comorbidities (diabetes mellitus, hypertension, hypercholesterolemia and prior statins).*

*-Model 4: Model 3 + angiographic severity (DS%, lesion length).*

Hct: hematocrit; Hgb: hemoglobin; RBC: red blood cells count.

**Table 4:** Coefficients for Hct model 4.

Model 4	Unstandardized Coefficients		Standardized Coefficients	t	*P* Value	95.0% Confidence Interval for B			
	B	Std. Error	Beta			Lower Bound	Upper Bound	R	R square
(Constant)	1.631	0.574		2.840	0.005	0.503	2.758	0.324	0.105
Hct	0.019	0.005	0.159	3.759	**<0.001**	0.009	0.028		
MCH	0.019	0.011	0.064	1.702	0.089	−0.003	0.041		
PLT	0.000	0.000	−0.036	−0.898	0.369	−0.001	0.000		
PDW	−0.014	0.017	−0.029	−0.784	0.433	−0.048	0.020		
WBC	−0.008	0.010	−0.032	−0.821	0.412	−0.028	0.011		
Age	0.000	0.002	−0.005	−0.121	0.904	−0.005	0.004		
Sex	0.020	0.053	0.016	0.378	0.706	−0.083	0.123		
BMI	0.006	0.007	0.032	0.858	0.391	−0.007	0.019		
Diabetes-Mellitus	−0.007	0.045	−0.006	−0.161	0.872	−0.097	0.082		
Hypertension	−0.012	0.046	−0.010	−0.264	0.792	−0.102	0.078		
Hyperchole-sterolemia	−0.088	0.044	−0.074	−1.982	0.048	−0.175	−0.001		
Prior Statins	−0.072	0.044	−0.061	−1.630	0.104	−0.159	0.015		
DS%	−0.001	0.003	−0.016	−0.444	0.657	−0.007	0.004		
Lesion length (mm)	−0.009	0.001	−0.244	−6.806	**<0.001**	−0.012	−0.006		

BMI: body mass index; DS%: diameter stenosis percentage; Hct: hematocrit; MCH: mean corpuscular hemoglobin; PDW: platelet distribution width; PLT: platelets; WBC: white blood cells.

Due to multicollinearity, principal component analysis (PCA) was performed for RBC parameters (Hb, Hct, MCV, RDW) and hematologic covariates (WBC, PLT) using Varimax rotation (eigenvalue >1). All retained variables had VIF < 3 in final models, confirming acceptable collinearity.

### Subgroup and sensitivity analyses

- **Diabetes Status**: The Hct-IMR association remained significant in both diabetic (β = 0.021, *P* = 0.007) and non-diabetic subgroups (β = 0.017, *P* = 0.012), suggesting robustness across metabolic states.
- **Sensitivity Analysis**: Exclusion of extreme Hct outliers (30–50%) did not attenuate the Hct-IMR relationship (β = 0.020, *P* < 0.001).
- **Vessel-Specific Effects**: Initial comparison between LAD (LAD n = 288) and non-LAD (LCx n = 250, RCA n = 195) vessels revealed divergent associations for Hct, with significant correlations in non-LAD territories (β = 0.022, *P* < 0.001) but not in LAD lesions (β = 0.008, *P* = 0.357). Subsequent vessel-specific analyses incorporating all RBC parameters demonstrated this pattern held consistently across measures. The LCx showed the strongest associations (Hct: β = 0.021, *P* = 0.010; Hgb: β = 0.007, *P* = 0.007; RBC: β = 0.193, *P* = 0.014), followed by the RCA (Hct: β = 0.019, *P* = 0.031; Hgb: β = 0.006, *P* = 0.017; RBC: β = 0.188, *P* = 0.023), while LAD lesions showed no significant relationships for any parameter (Table 5). This graduated response pattern suggests microcirculatory resistance in non-anterior territories may be particularly sensitive to hematologic determinants, with the LCx demonstrating the greatest dependence on RBC parameters.

**Table 5:** Regression results for all the in different variables in each’s model 4 linear regression.

Variables	LAD	LCx	RCA
**RBC**	.089 (−0.075 to 0.252) *P* = 0.288	.193 (0.039 to 0.347) ***P* = 0.014**	.188 (0.026 to 0.350) ***P* = 0.023**
**Hct**	.008 (−0.009 to 0.025) *P* = 0.357	.021 (0.005 to 0.037) ***P* = 0.010**	.019 (0.002 to 0.036) ***P* = 0.031**
**Hgb**	.003 (−0.002 to 0.008) *P* = 0.260	.007 (0.002 to 0.011) ***P* = 0.007**	.006 (0.001 to 0.011) ***P* = 0.017**

Hct: hematocrit; Hgb: hemoglobin; LAD: left anterior descending; LCx: left circumflex; RCA: right coronary artery; RBC: red blood cells count.

## Discussion

Our study investigated the associated between the IMR and RBC parameters, demonstrating that higher levels of all of the primary RBC parameters (RBC count, Hct and Hgb) are independently associated with higher IMR even after adjusting for confounders that might be considered as conventional risk factors for microvascular dysfunction. The stability of our results after sequential adjustments for potential confounders, combined with the consistency across all primary RBC parameters, plays a key role in reinforcing the independent impact of RBC parameters on microcirculatory resistance.

### Concordance with previous studies

Recent literature has increasingly highlighted the association between RBC parameters and cardiovascular disease, particularly following the landmark TAMRISK study [19], which demonstrated in its 28-year follow-up that borderline polycythemia was linked to higher coronary heart disease (CHD) mortality. Subsequent studies have further explored this relationship, showing that elevated Hct and Hgb levels correlate with acute myocardial infarction (AMI) mortality in women [37], as well as MI risk and CHD mortality in men [19,20]. Additionally, a U-shaped association has been reported between these RBC parameters and adverse outcomes in CHD and heart failure [21–24,38,39]. Case reports have also documented correlations between increased RBC mass in polycythemia vera and conditions such as MI, heart failure [40], ST-elevation myocardial infarction (STEMI) [41], and cardiomyopathy [42,43]. Our findings align with and further substantiate this growing body of evidence, reinforcing the role of RBC parameters in microcirculatory dysfunction and cardiovascular pathology.

RBC parameters serve as indirect markers of blood viscosity, which plays a critical role in microcirculatory hemodynamics. Elevated viscosity increases vascular resistance, impairing tissue perfusion—a phenomenon demonstrated in radiographic contrast media studies, where higher-viscosity formulations significantly reduced capillary flow compared to lower-viscosity agents [44]. Massive sludging can be found in diseases that cause erythrocyte aggregates such as diabetes mellitus [45]. The clinical relevance of these mechanisms is underscored by case reports, such as that of an 85-year-old patient with congestive heart failure (CHF) and stroke, in whom elevated blood viscosity correlated with adverse outcomes [46]. Collectively, these observations suggest that RBC-driven increases in viscosity may contribute to microvascular impairment through altered rheology, endothelial shear stress, and oxygen delivery—a pathway consistent with our findings of heightened microcirculatory resistance in patients with elevated RBC parameters.

### Vessel-specific heterogeneity

The observed differences in microvascular dysfunction between the LAD, LCx, and RCA likely stem from distinct hemodynamic and anatomical characteristics. The LAD benefits from higher endothelial shear stress, which confers atheroprotective effects, while the LCx exhibits greater molecular viscosity and the RCA demonstrates the highest wall stress [47]. Furthermore, the increased tortuosity typically seen in the RCA and LCx subjects these vessels to greater mechanical strain, elevating flow resistance. This is supported by previous studies showing that tortuous coronary arteries experience reduced perfusion pressure [48], diminished flow rates [49], and decreased blood pressure [50]. Notably, coronary resistance can increase by up to 92% in tortuous segments during exercise [51], and severe tortuosity has been associated with impaired myocardial blood flow reserve [52]. These factors collectively can explain the regional variations in microvascular dysfunction observed in our study.

### Implications

The observed association between RBC parameters and impaired microvascular function may have particular relevance for MINOCA/INOCA patients. Our findings suggest that the routine complete blood count, which imposes zero extra cost or procedural burden, could be studied as a first-line tool to help identify patients at heightened risk for coronary microvascular dysfunction. Beyond screening, this mechanistic link—between erythrocyte parameters and microcirculatory resistance—suggests that hemorheology may represent a novel therapeutic axis to explore. In conditions characterized by elevated hematocrit (e.g., polycythemia vera), therapeutic phlebotomy is a standard intervention to reduce viscosity and thrombotic risk. While our study population had hematocrit levels mostly within the normal range, the linear relationship we observed generates a testable hypothesis that therapeutic modulation of hemorheology—or the influence of modifiable lifestyle factors upon it—could improve coronary microvascular function, warranting investigation in prospective interventional trials.

### Strengths and limitations

The study’s large cohort of lesions and the consistent associations observed across all primary erythrocyte parameters—even after comprehensive adjustment for confounders—strengthen the validity of our findings. These results align with established evidence linking blood viscosity to microcirculatory impairment and extend prior observations on erythrocyte-related cardiovascular outcomes. Together, these data provide new insights into the relationship between hematologic parameters and microvascular dysfunction, particularly in non-LAD territories.

Our study has several limitations. First, angiography-derived IMR, while validated, is not the invasive wire-based gold standard. Second, hematologic parameters were measured at a single time point, are subject to plasma volume variation, and lack data on specific etiologies (e.g., polycythemia vera, CKD severity) or erythropoietic activity markers. Third, the cohort’s predominantly Asian ethnicity and exclusive focus on intermediate coronary lesions may limit generalizability. Fourth, comorbidities were recorded as present/absent without data on severity, duration, or control. Fifth, while statistically significant and consistent, the observed correlations were modest in magnitude (e.g., Hgb: r = 0.172), congruent with CMD’s multifactorial nature, and should be interpreted as identifying one significant component rather than a dominant driver. Finally, the absence of established clinical cutoff values challenges direct translation into practice.

Future studies integrating a broader panel of hemodynamic, inflammatory, and hematologic markers with serial measurements are warranted to build more comprehensive models and test therapeutic hypotheses.

## Conclusion

In this study, red blood cell count, hematocrit, and hemoglobin demonstrated independent positive associations with elevated coronary microcirculatory resistance. These findings suggest that elevated erythrocyte parameters may contribute to the pathogenesis of coronary microvascular dysfunction and represent a potential therapeutic target for improving microcirculatory function.

### Impact on daily practice

Our results identify erythrocyte parameters as novel, independent predictors of coronary microcirculatory resistance, offering a hematologic lens for evaluating microvascular dysfunction, particularly in MINOCA/INOCA cases. Routine blood measures like hematocrit and hemoglobin may therefore serve as practical biomarkers to improve risk stratification and enable personalized management. We recommend integrating RBC assessment into standard clinical practice to better identify high-risk individuals and inform potential treatment approaches focused on modulating blood viscosity or flow properties, while also highlighting the need for further research to validate causal mechanisms and assess the efficacy of viscosity-modifying interventions.

## Supporting information

S1 Table The population data. (XLSX)

S2 Table Coefficients for Hct model 4 with Leukocyte Subtypes. (DOCX)

## Abbreviations

CMD: coronary microvascular dysfunction
Angio-IMR: angiography-derived index of microcirculatory resistance
IMR: index of microcirculatory resistance
QFR: quantitative flow ratio
mQFR: monoplane quantitative flow ratio
RBC: red blood cells count
Hct: hematocrit
Hgb: hemoglobin
CBC: complete blood count
MCV: mean corpuscular volume
MCH: mean corpuscular hemoglobin
MCHC: mean corpuscular hemoglobin concentration
RDW CV: red cell distribution width - coefficient of variation
RDW SD: red cell distribution width - standard deviation
PLT: platelets
PDW: platelet distribution width
WBC: white blood cells
DS: diameter stenosis
LAD: left anterior descending
LCx: left circumflex
RCA: right coronary artery.
