# Supplementary material for: A hemorheological perspective on coronary microvascular dysfunction: Association of erythrocyte parameters with angiography-derived coronary microcirculatory resistance
Source: PLoS One. 2026 Mar 25;21(3):e0345562. doi: 10.1371/journal.pone.0345562 (PMC13016340; doi:10.1371/journal.pone.0345562)
Supplement: S2 Table — (DOCX) [file pone.0345562.s002.docx]

| Table S. Coefficients for Hct model 4 with Leukocyte Subtypes | | | | | | | | | | | |
| --- | --- | --- | --- | --- | --- | --- | --- | --- | --- | --- | --- |
| Model | | Unstandardized Coefficients | | Standardized Coefficients | t | *P* Value | 95.0% Confidence Interval for B | | Collinearity Statistics | |  |
|  |  | B | Std. Error | Beta |  |  | Lower Bound | Upper Bound | Tolerance | **VIF** |  |
| 1 | (Constant) | 1.839 | .580 |  | 3.172 | 0.002 | .701 | 2.978 |  |  |  |
|  | HCT | .017 | .005 | .148 | 3.361 | **0.001** | .007 | .027 | .650 | 1.539 |  |
|  | MCH | .018 | .011 | .060 | 1.587 | 0.113 | -.004 | .040 | .883 | 1.132 |  |
|  | PLT | .000 | .000 | -.034 | -.822 | 0.411 | -.001 | .000 | .727 | 1.375 |  |
|  | PDW | -.019 | .017 | -.040 | -1.095 | 0.274 | -.054 | .015 | .926 | 1.080 |  |
|  | WBC | .072 | .066 | .281 | 1.100 | 0.272 | -.057 | .201 | .019 | 51.650 |  |
|  | LYMPH | -.055 | .077 | -.053 | -.716 | 0.474 | -.207 | .096 | .227 | 4.402 |  |
|  | MONO | -.298 | .175 | -.085 | -1.698 | 0.090 | -.643 | .046 | .505 | 1.978 |  |
|  | NEUT | -.071 | .067 | -.255 | -1.072 | 0.284 | -.202 | .059 | .022 | 44.931 |  |
|  | EO | .311 | .191 | .066 | 1.629 | 0.104 | -.064 | .686 | .765 | 1.308 |  |
|  | BASO | -1.686 | .918 | -.070 | -1.836 | 0.067 | -3.489 | .117 | .855 | 1.170 |  |
|  | Age | .000 | .002 | -.002 | -.055 | 0.956 | -.005 | .005 | .699 | 1.430 |  |
|  | Sex | .027 | .054 | .021 | .490 | 0.625 | -.080 | .134 | .688 | 1.454 |  |
|  | BMI | .005 | .007 | .027 | .719 | 0.472 | -.008 | .018 | .872 | 1.146 |  |
|  | DMs | -.001 | .046 | -.001 | -.015 | 0.988 | -.090 | .089 | .927 | 1.079 |  |
|  | HTN | -.019 | .046 | -.016 | -.417 | 0.677 | -.109 | .071 | .898 | 1.113 |  |
|  | Hypercholesterolemia | -.083 | .044 | -.070 | -1.866 | 0.062 | -.170 | .004 | .899 | 1.113 |  |
|  | Prior Statins | -.081 | .044 | -.069 | -1.825 | 0.068 | -.168 | .006 | .880 | 1.136 |  |
|  | DS% | -.001 | .003 | -.020 | -.541 | 0.589 | -.007 | .004 | .967 | 1.034 |  |
|  | Lengh | -.009 | .001 | -.245 | -6.856 | **<0.001** | -.012 | -.006 | .982 | 1.019 |  |
| a. Dependent Variable: Simulated Hyperemic MR | | | | | | | | | | | |
